# BTFormer: Blast transformer for human blastocyst components segmentation

**DOI:** 10.1371/journal.pone.0328919

**Published:** 2025-08-22

**Authors:** Hua Wang, Yiming Li, Linwei Qiu, Jicong Zhang, Jingfei Hu

**Affiliations:** 1 School of Biological Science and Medical Engineering, Beihang University, Beijing, China; 2 Hefei Innovation Research Institute, Beihang University, Hefei, China; 3 School of Medical Informatics Engineering, Anhui University of Traditional Chinese Medicine, Hefei, China; King Saud University/Zagazig University, EGYPT

## Abstract

Assessing embryo quality through segmentation of blastocyst components is crucial, as embryo morphology directly correlates with its potential for implantation. However, automatic blastocyst segmentation remains a challenging task due to factors such as poor contrast, noise, and ambiguous boundaries between different tissue structures. In this study, we introduce a novel transformer-based architecture, termed BTFormer (Blastocyst Transformer), designed to effectively segment blastocyst components. Firstly, we use an axial-free attention mechanism with lower computational resources, which catches non-local feature maps with long-range cues to alleviate the mistake of local structure. Secondly, to enjoy the rotation consistency of the embryo images, we propose an axial-free attention block with a soft aggregation operation to embed features extracted by axial-free attention with different angles, which collect global cues and broadcast a diversified receptive field. We validated our method on a typical public dataset and achieved the state-of-the-art segmentation performance with accuracy, precision, recall, Dice coefficient, and Jaccard index of 93.86%, 91.81%, 92.25%, 92.02% and 85.45%. Extensive qualitative experimental results demonstrate the effectiveness of our proposed method.

## Introduction

Infertility is one of the most pressing health issues worldwide [1]. It is characterized by the inability to achieve conception through regular unprotected sexual intercourse [2].

In vitro fertilization (IVF) is a prevalent approach to addressing infertility [3]. During IVF, embryos are cultured in a controlled laboratory setting in vitro until they reach the blastocyst stage. Subsequently, a human blastocyst exhibiting high implantation potential is selected for transfer by visual evaluation of its morphological characteristics. This selection process relies on the evaluation of the inherent morphological structures, including cell density, size, and expansion level, to grade embryo quality [4]. However, such evaluations involving human intervention may introduce intra- and inter-variability. In addition, IVF is labor intensive, error-prone and requires specialized domain knowledge. Consequently, automatic segmentation of distinct tissue regions in blastocyst images becomes imperative to facilitate the quality assessment of embryos.

**Fig 1:**
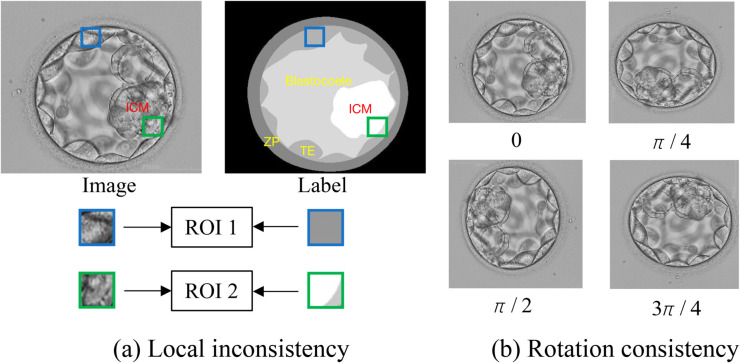
Two characteristics we discover in blastocyst images. (a) Local inconsistency. The blue box and green box represent two ROIs (Regions of Interest). Two different types of tissues may share similar textures in one local region. (b) Rotation consistency. Embryo images do not lose semantic information when rotated around the center at any angle.

In blastocyst images, one of the most challenging characteristics observed is local inconsistency. These images are captured using the Hoffman optical sampling system, known for its three-dimensional relief effect and transparent tissue properties. The presence of overlapping structures in the image can contribute to this inconsistency. Moreover, under low-light conditions, the resulting low contrast and noise further exacerbate this phenomenon. As depicted in Fig 1(a), two small regions of interest (ROI 1 and ROI 2) are extracted from an embryo image, with their locations marked both in the original image and its corresponding label image. In particular, ROI 1 and ROI 2 exhibit similar colors and textures, despite containing different classes in their label images. This observation suggests that the categorization of a particular area is influenced not only by its colors and textures but also by its spatial relationship with neighboring regions, indicating the importance of contextual relationships in embryo images. And the main labels of a human blastocyst, as illustrated in the label of Fig 1(a), typically include background, inner cell mass (ICM), blastocoele, trophectoderm (TE), and zona pellucida (ZP). Specifically, TE is typically encased by ZP and blastocoele from two sides, while ICM tends to be located within the boundaries of TE, closer to the center of the embryo [5]. Furthermore, blastocyst images are commonly captured under low-light conditions, despite the fact that the entire embryo is transparent and has good luminosity. Consequently, determining the edge, particularly in a localized view such as ROI 2 in Fig 1(a), becomes problematic. In summary, the combination of local inconsistency and blurred edges necessitates the incorporation of contextual and non-local information for accurate analysis.

To mitigate these challenges and improve blastocyst segmentation accuracy, we introduce a novel standalone transformer-based network termed BTFormer. At the heart of our approach lies an axial-free attention mechanism, devised to decompose 2D attention into a single 1D attention in any direction. This mechanism enables the capture of long-range dependencies while maintaining computational efficiency. Additionally, we enhance the positional terms to be context-dependent and gradient-dependent, imbuing our attention mechanism with position sensitivity and thereby enhancing performance with minimal additional computational costs.

**Fig 2:**
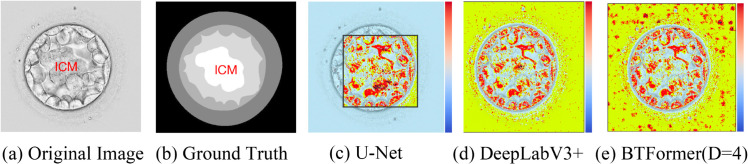
Effective receptive fields (colored dots) of different models on a 256×256 input image. It is a receptive field estimation based on non-negligible gradients and reveals global interactions to some extent. We have dispersed non-local information across the whole image. *D* is a parameter of our BTFormer.

Conversely, our axial-free attention factorization compromises global interactions and constrains the receptive field, critical for pixel-level tasks such as segmentation, particularly with high-resolution inputs [6]. As illustrated in Fig 2(c), although U-Net integrates multiple scale features, it still lacks comprehensive global interaction, as evident from the effective receptive field measurement. This metric serves as a tangible indicator of the amount of information a model can effectively leverage [7]. Strategies such as dilated convolutions, employed in Blast-Net [5], and DeepLabV3+ [8], may mitigate this deficiency, as depicted in Fig 2(d). To enhance global information integration, we propose a novel axial-free attention block that combines our axial-free attention mechanism with an additional inductive bias, namely, *rotation consistency*, as depicted in Fig 1(b). We observe that semantic information in embryo images remains preserved when the images are rotated around their center at any angle. This stands in contrast to certain natural images, such as handwritten digits, where a ‘6’ might be erroneously perceived as a ‘9’ when rotated by 180 degrees. In essence, a model can effectively capture semantic information from any orientation in blastocyst images. Consequently, our axial-free attention block comprises several parallel yet interconnected axial-free attention layers set at specific axial angles, enabling the learning of diverse representations from multiple directions. To amalgamate information from all orientations, we employ a specialized soft aggregation operation to consolidate various representations into a final feature map.

In the end, we construct an encoder-decoder architecture called blast-transformer (BTFormer) by substituting conventional convolution blocks with our proposed axial-free attention blocks. This modification yields a substantially broader receptive field, as illustrated in Fig 2(e). We assess the performance of our model using a publicly available embryo dataset for segmentation. The experimental results affirm the efficacy and superiority of our approach.

In summary, the main contributions of this paper are as follows:

- We introduce a novel standalone transformer-based network named BTFormer aimed at enhancing blastocyst segmentation performance while maintaining computational efficiency.
- We devise a straightforward yet efficient axial-free attention mechanism to capture non-local contextual information from any direction while ensuring computational efficiency. Moreover, we introduce learnable sinusoidal relative positional encoding to enhance the positional relationships between elements within the blastocyst image, thereby further enhancing segmentation performance.
- Building upon the axial-free attention mechanism, we introduce a novel axial-free attention block featuring a soft aggregation operation designed to gather global representations, thereby significantly enriching the receptive field.

The remaining sections are structured as follows. The Related works section provides an overview of related works concerning blastocyst segmentation, semantic segmentation, and transformer architectures. In the Methodology section, we detail the axial-free attention mechanism and introduce our proposed model, BTFormer. The Materials section outlines the dataset utilized in the experiments, along with evaluation metrics and implementation details. The experimental results are presented in the Experimental results section. Finally, the Conclusions section concludes the paper and suggests potential avenues for future research.

## Related works

### Blastocyst segmentation

In traditional computer vision, many tasks also attempt to automatically segment blastocyst images. For example, initial attempts mainly use the traditional method (Level set) to segment inner cell mass and trophectoderm [9]. By correcting the curve, based on the level set method, the above problems are alleviated and some improvements are made [10]. Identifying ICM boundaries through text information (Gabor and DCT features) combined with the level-set method can enhance the expression of features to a certain extent [11]. By introducing the idea of clustering, it is possible to better aggregate the features of the same organization [12]. However, in clinical practice, the morphology of blastocysts is diverse, making it difficult to effectively distinguish different tissues through initial localization, and hence requires human intervention.

With the development of deep learning, some studies attempt to combine traditional methods with deep learning for the recognition of blastocyst tissue, such as extracting traditional features and sending them into the two-layer neural network to identify ZP, TE, and ICM [13]. However, this simple combination effect is not ideal. Therefore, pure end-to-end deep learning methods are applied in this field, such as using a Fully Convolution Neural (FCN) network to segment the ICM [14]. The combination of dialed convolution and U-Net has also achieved good results [15]. Recently, some methods have also utilized multi-scale extraction of blastocyst image features to identify different tissues, which has improved the accuracy of tissue recognition to some extent [16]. I2CNet method [17] explored the relationship between classes and improved boundary recognition performance. However, these methods increase the perception field of a single pixel but do not alter the attribute relationship between the pixels.

### Semantic segmentation

Deep learning-based semantic segmentation algorithms have garnered considerable attention due to their effectiveness [18]. FCN [19] is the first convolutional neural network (CNN) designed for image segmentation, demonstrating the possibility of deep networks for semantic segmentation in an end-to-end manner. This fully convolutional architecture of stacking convolution layers is fairly simple with the limitations of lacking global context information. Subsequently, many frameworks have been proposed and strengthened both in the depth and width of networks. One of the most popular approaches is the encoder-decoder architecture, where the resolution of the features changes with the process of encoding and decoding in DeConvNet [20], SegNet [21], LinkNet [22], and W-Net [23]. Meanwhile, HRNet [24] retains high-resolution representations in the whole encoding process by linking to the different resolution streams parallel. Another typical network is based on multi-scale analysis, where feature pyramids are deployed. Feature Pyramid Network (FPN) [25]) was one of the most prominent networks, which was developed with a bottom-up pathway, a top-down pathway, and lateral connections to fuse the features of different scales. Pyramid Scene Parsing Network (PSPNet [26]) was proposed to enrich the global contexts by a pyramid pooling module. In particular, DeepLabV3+ [8] is an encoder-decoder architecture with atrous separable convolution, where the DeepLabV3 framework serves as the encoder. Attention mechanisms are also applied to computer vision, which is to teach deep networks to ignore irrelevant information and focus on important information. Most studies focus on the use of masks to form an attention mechanism, which is to identify the key features in the image through learned weight [27,28].

There are several models initially developed for medical or biomedical image segmentation, which are inspired by FCNs and encoder-decoder models. Ronneberger *et al*. [29] proposed U-Net for segmenting biological microscopy images. After then, various U-Net variants have been proposed for medical images analysis, such as Attention U-Net [30], Unet++ [31], DU-Net [32], PraNet [33], nnUnet [34] and so on. These networks can be applied to blastocyst image segmentation, but they are not designed specifically for embryo data, their performance on embryo datasets is limited. Moreover, these methods only exploit efficient locality at the cost of losing long-range cues.

### Transformer

Vaswani *et al*. [35] originally proposed the transformer to learn long-range dependencies between the input tokens in natural language processing (NLP) tasks. From then on, models (like GPT families [36–38], BERT [39] and its variants [40,41]) based on transformers have gradually emerged as predominant methods in NLP problems [42,43]. These successful NLP applications stimulate the attempt of adopting transformers to computer vision areas. Dosovitskiy *et al*. [44] proposed a novel Vision Transformer (ViT) network, obtaining competitive results compared with the current pure CNN-based networks in a classification task. MaX-Deeplab [45] was designed for panoptic segmentation based on transformers. Segmentation Transformer (SETR) [46] applies a transformer as an encoder and obtains competitive results of semantic segmentation. TransUNet [47] was developed to combine the strength of transformers and U-Net, which behaves well in the medical image segmentation task. A novel Squeeze-and-Expansion transformer was classified in Segtrans [48] to maintain the effective receptive fields at high feature resolutions. MedT [49] was designed to overcome the few data samples in medical imaging, as transformers are usually trained on a large-scale dataset. As is commonly understood, self-attention excels at capturing long-range contextual dependencies within the feature map, in contrast to the localized interactions typically facilitated by convolutions. However, it increases the computation complexity to $𝒪\left(H^{2}W^{2}\right)$ with height *H*, weight *W*, thus becoming extremely expensive, especially for a feature map of large size. Moreover, it does not parlay any positional information, which is of great importance in vision fields. To mitigate the heavy computation cost, local memory block ${𝒩}_{k\times k}\left(a\right)$ with the local k×k square region centered around each location $a=(x_{i},y_{i})$ is proposed [50], which serves as a bank for calculating the output. This local constraint indeed reduces the computational cost to $𝒪\left(HWk^{2}\right)$ when *k* is small enough, whereas it sacrifices the non-local representations.

However, despite the utilization of CNN-based and transformer-based approaches for segmentation in human blastocyst images, two primary challenges persist: 1) How to effectively capture non-local dependencies and alleviate local inconsistency, as depicted in Fig 1(a), in order to address the intricate manifestations of embryo morphology, particularly concerning blurred edges and similar textures. 2) How to integrate global contextual features and attain a broad receptive field to enhance the network’s learning capacity. To tackle these challenges, we propose a novel Blast-transformer (BTFormer) architecture based on the axial-free attention layer and axial-free attention blocks. We introduce an axial-free attention mechanism capable of capturing non-local information from any direction with reduced computational cost. Furthermore, the axial-free attention block capitalizes on the principle of rotation consistency in blastocyst images to extract global salient information effectively.

## Methodology

In this section, we begin by presenting the overall construction of BTFormer. Subsequently, we introduce the principal component of BTFormer, which is the axial-free attention block. Finally, we provide a detailed description of the mechanism employed within the axial-free attention block.

### BTFormer

**Fig 3:**
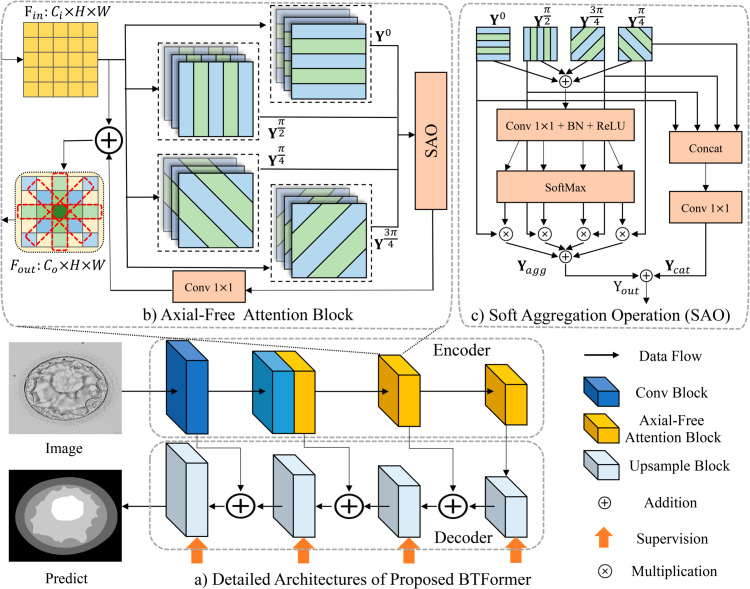
An overview architecture of BTFormer. a) Detailed Architectures of Proposed BTFormer. It extracts global features with a transformer-based backbone as an encoder. The decoder consists of upsampling blocks, comprising an upsampling operator and some simple convolutional layers (Conv-BN-ReLU). b) Axial-free Attention Block (D = 4). After passing through this module, the features will aggregate the four representative axial features from different angles and combine them with the original feature to form output features, which include global information (red dashed boxes represent the features of 0, 45, 90 and 135 degrees respectively). c) Soft aggregation operation. The left branch $Y_{agg}$ can condense biased information into one output keeping the same size and generating weighted and diversified features. $Y_{cat}$ is a simple concatenate operation to facilitate feature fusion.

We propose a BTFormer for the semantic segmentation of embryo images. It is an encoder-decoder structure combining multi-scale fusion as exhibited in Fig 3(a), which retains a new stand-alone transformer-based encoder by replacing the original convolution blocks with our proposed axial-free attention blocks. In a word, attention in our BTFormer serves as a stand-alone primitive rather than being parlayed on top of convolutions. Due to the high resolution and its heavy costs concerning the transformer, the first layer and the second layer comprise the traditional convolution blocks to extract image features. The role of the convolution blocks is to reduce the computation and resolution of feature maps. Note that the second convolution block does not change the resolution of feature maps as there is no pooling operation at the end of it. The resolution changes after every axial-free attention block because an additional 2×2 max pooling operator is added to all attention blocks.

### Axial-free attention block

The axial-free attention (described in section Axial-free attention mechanism) captures long-distance dependencies. To facilitate a wider receptive field, we globally collect widespread information by applying rotation consistency. It refers to an observation that embryo images retain physiological significance no matter how many degrees you rotate them, as seen in Fig 1(b). In other words, semantic cues from any direction count for segmentation. Thus, we arrange several axial-free attention layers uniformly spaced around the whole directions in a 2D space:

D×¯θ=π, (1)

where *D* is the number of axial-free attention layers we employ, and ¯θ denotes the angle between two adjacent layers. The selection of *D* will be discussed in the experimental section. We choose a parallel structure with *D* = 4, followed by a soft aggregation operation, as shown in Fig 3(c). We proposed an axial-free attention block that helps globally extract context information, as depicted in Fig 3(b).

As described in Fig 3(b) in the setting of *D* = 4, we apply axial-free attention to obtaining semantic information in four directions parallel: $Y^{0},Y^{\frac{\pi }{4}},Y^{\frac{\pi }{2}},Y^{\frac{3\pi }{4}}$, where the angle $¯\theta =\frac{\pi }{4}$ by Eq 1. Multi-head attention is used in every axial direction and the results of each head are concatenated as the output. The whole block employs a residual structure while two 1×1 convolutions in the residual branch are kept to shuffle the features. Specifically, our axial-free attention block in Fig 3(c) can collect semantic information at 0, 45, 90, and 135 degrees by four axial-free attention layers, thus strongly broadcasting diversified cues with a global but sparse way in Fig 3(b). Note that ¯θ can be exaggerated or narrowed by adjusting the number *D*. This design enriches context information from every direction and promotes a global receptive field, which will be proved in the next section. Moreover, our block even obtains rotation invariant to some extent. When the input is rotated by multiples of ¯θ, the output shares global connections from the same directions.

For each point of feature maps, the required semantic information is not always the same. The effect may not be suitable if we simply add these four axial-free attention results. As shown in Fig 3(c), we propose a fusion operation inspired by the Split Attention [51]. Assume that the axial-free attention block consists of *D* output of the axial-free attention: $Y^{\left(d\right)},d=1,...,D$. The final results $Y_{out}$ are obtained by the sum of two branches *i.e.* $Y_{agg}$ and $Y_{cat}$ with the same inputs $Y^{d}$

$$Y_{out}=Y_{agg}+Y_{cat}.$$ (2)

For the left branch in Fig 3(c), it outputs *D* sets of contextualized features, which are then aggregated into one set using an aggregation operation

$$\begin{array}{c} A^{\left(d\right)}={\text{Conv}}^{\left(d\right)}\left(\text{sum}\left(Y^{\left(d\right)}\right)\right),d=1,...,D,\\ Y_{agg}=\text{softmax}\left(A^{\left(1\right)},...,A^{\left(D\right)}\right)\left(Y^{\left(1\right)},...,Y^{\left(D\right)}\right), \end{array}$$ (3)

where Conv denotes the convolutional layers. Here, the weights of each axial-free attention layer are learned by the convolutions with the sum of each axial-free attention layer, and taking softmax in all the directions. The final aggregation $Y_{out}$ of *D* axial-free attention layers are aggregated by a weighted sum over each axial-free attention layer.

For the right branch in Fig 3(c), it is a simple concat operation Cat with a 1×1 convolutional layer Conv1×1 using the following equation

$$Y_{cat}=\text{Conv1}\;\times \;1(\text{Cat}[Y^{\left(1\right)},...,Y^{\left(D\right)}]).$$ (4)

### Axial-free attention mechanism

**Fig 4:**
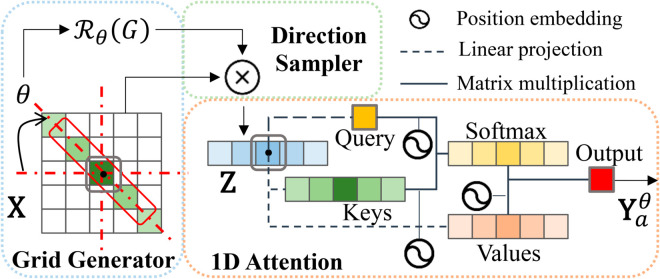
The structure of axial-free attention. The combination of the grid generator, direction sampler, and 1D attention defines our axial-free attention mechanism. For every pixel of input feature map **X**, it outputs a final result **Y** based on a whole image view *i.e.* direction feature map **Z** which varies with different directions *θ*.

We propose a new attention mechanism called axial-free attention to extracting non-local correlation from any given direction at a low cost of computation. As depicted in Fig 4, the axial-free attention mechanism comprises four main components: the grid generator, direction sampler, 1D attention module and learnable sinusoidal relative positional encoding. For simplicity, here we describe single-head attention, however, it can be generalized to multi-headed attention as shown in experiments.

**Grid generator:** The role of the grid generator is to compute the pixel-wise location of the direction feature map **Z** for a given input $𝐗\in {\mathbb{R}}^{C_{i}\;\times \;H\;\times \;W}$, which is inspired from spatial transformer networks [52]. Different from the width axis and the height axis, the overlap areas between a view of other directions and the input feature change with various positions. For example, we will gain a larger field of view when we get close to the center point. In contrast, the field will turn narrow if we approach the corner points for a given direction *θ*. Thus, we project the direction feature map along the width or height axis and restrict its size (excluding the channel) to be equal to the width 1×W or height H×1 of an input feature map. In other words, the bank for computation is determined. Although some pixels are newly calculated and some other pixels are discarded, this projection can even improve the robustness.

In general, a pixel $a=(x_{i},y_{i})$ in the direction feature map is transformed on the original input grid $G=\{G_{i}^{\prime }\}$ where $G_{i}^{\prime }=(x_{i}^{\prime },y_{i}^{\prime })$. In this rotation case, the pixel-wise projection is

$$(\begin{array}{c} x_{i}\\ y_{i} \end{array})={ℛ}_{\theta }(G_{i})=[\begin{array}{cc} \cos\theta & -\sin\theta \\ \sin\theta & \cos\theta \end{array}](\begin{array}{c} x_{i}^{\prime }\\ y_{i}^{\prime } \end{array}),\theta \in [0,\pi ),$$ (5)

where $\left(x_{i}^{\prime },y_{i}^{\prime }\right)$ are the coordinates defining the sample grid in the input feature map. From Eq 5, we can control the direction feature map by setting the original input grid.

**Direction sampler:** The direction sampler can produce the sampled direction feature map **Z** by utilizing the sampling grid along with the input feature map **X**. This is achieved by applying a sampling kernel for any pixel $a=(x_{i},y_{i})$ in the direction feature map as:

$${𝐙}_{a}=\sum_{n}^{H}\sum_{m}^{W}{𝐗}_{nm}\Phi_{x}(x_{i}-m)\Phi_{y}(y_{i}-n),$$ (6)

where $\Phi_{x}$ and $\Phi_{y}$ define the interpolation (such as bilinear, and bicubic). Note that here we keep the spatial invariant between channels because the sampling in Eq 6 is performed independently for each channel.

Specifically, we use a bilinear interpolation, giving

$${𝐙}_{a}=\sum_{n}^{H}\sum_{m}^{W}{𝐗}_{nm}\max(0,1-|x_{i}-m|)\max(0,1-|y_{i}-n|).$$ (7)

**1D attention:**After obtaining the direction feature map **Z**, the final output **Y** can be calculated by 1D self-attention. To be concrete in Fig 4, the axial-free attention along the given parameter of an angle *θ* is defined as follows:

$${𝐘}_{a}^{\theta }=\sum_{b\in {𝒩}_{H\;\times \;W}\left(a,\theta \right)}{\text{softmax}}_{b}({𝐐}_{a}^{T}{𝐊}_{b}+{𝐐}_{a}^{T}{𝐑}_{b-a}^{Q}\;+\;{𝐊}_{b}^{T}{𝐑}_{b-a}^{K})\left(V_{b}+R_{b-a}^{V}\right),\theta \in [0,\pi ),$$ (8)

where *θ* is the axial angle between the axis and the width axis. Here, queries ${𝐐}_{a}={𝐖}_{Q}{𝐙}_{a}$, keys ${𝐊}_{b}={𝐖}_{K}{𝐙}_{b}$, and values ${𝐕}_{b}={𝐖}_{V}{𝐙}_{b}$ are linear transformations of the pixel in the direction map, where ${𝐖}_{Q},{𝐖}_{K},{𝐖}_{V}\in {\mathbb{R}}^{C_{o}\;\times \;C_{i}}$ are learnable as the same with the settings of self-attention. Moreover, ${𝐑}^{Q}$, ${𝐑}^{K}$, and ${𝐑}^{V}$ are learnable sinusoidal relative positional encoding, which will be introduced later. Considering that the width of an input *W* is not always the same as the height *H*, we project the direction into the height axis when *θ* is more than $\frac{\pi }{2}$ to get a multi-range dependency, formulated as:

$${𝒩}_{H\;\times \;W}\left(a,\theta \right)=\left\{ \begin{array}{c} {𝒩}_{1\;\times \;W}\left(a,\theta \right),\theta \in [0,\frac{\pi }{2})\\ {𝒩}_{H\;\times \;1}\left(a,\theta -\frac{\pi }{2}\right),\theta \in [\frac{\pi }{2},\pi ) \end{array}\right..$$ (9)

Particularly, when θ=0, our equation is the same as the width-axis attention

$${𝐘}_{a}^{0}=\sum_{b\in {𝒩}_{H\;\times \;W}\left(a,0\right)}{\text{softmax}}_{b}({𝐐}_{a}^{T}{𝐊}_{b}+{𝐐}_{a}^{T}{𝐑}_{b-a}^{Q}\;+\;{𝐊}_{b}^{T}{𝐑}_{b-a}^{K})\left(V_{b}+R_{b-a}^{V}\right)\;=\sum_{b\in {𝒩}_{1\;\times \;W}\left(a\right)}{\text{softmax}}_{b}({𝐐}_{a}^{T}{𝐊}_{b}+{𝐐}_{a}^{T}{𝐑}_{b-a}^{Q}\;+\;{𝐊}_{b}^{T}{𝐑}_{b-a}^{K})\left(V_{b}+R_{b-a}^{V}\right).$$ (10)

When *θ* comes to $\frac{\pi }{2}$, the axial-free attention turns to the height-axis attention

$${𝐘}_{a}^{\frac{\pi }{2}}=\sum_{b\in {𝒩}_{H\;\times \;W}\left(a,\frac{\pi }{2}\right)}{\text{softmax}}_{b}({𝐐}_{a}^{T}{𝐊}_{b}+{𝐐}_{a}^{T}{𝐑}_{b-a}^{Q}\;+\;{𝐊}_{b}^{T}{𝐑}_{b-a}^{K})\left(V_{b}+R_{b-a}^{V}\right)\;=\sum_{b\in {𝒩}_{H\;\times \;1}\left(a\right)}{\text{softmax}}_{b}({𝐐}_{a}^{T}{𝐊}_{b}+{𝐐}_{a}^{T}{𝐑}_{b-a}^{Q}\;+\;{𝐊}_{b}^{T}{𝐑}_{b-a}^{K})\left(V_{b}+R_{b-a}^{V}\right).$$ (11)

By conducting our axial-free attention, we reduce the computation cost to $𝒪\left(H^{2}W\right)$ or $𝒪\left(HW^{2}\right)$. Meanwhile, it can capture long-distance dependencies instead of focusing on local interactions.

**Learnable sinusoidal relative positional encoding:** Position encoding provides cues to remedy information about spatial relationships. Additional position bias terms are usually added to values, which are often utilized to make the model sensitive to the positional information [50]. We propose learnable sinusoidal relative positional encoding (LSRPE), aiming to bring in the continuity bias with adaptability.

Given a pixel coordinate a=(i,j) and another pixel coordinate b=(l,n), our LSRPE is defined as:

$$R_{b-a}^{*}=\left[\sin\left(R_{l-i}^{*}\right)\;\sin\left(R_{n-j}^{*}\right)\right]\in {\mathbb{R}}^{C_{o}},*=Q,K,V,$$ (12)

where the learnable parameters $R_{l-i}^{*},R_{n-j}^{*}\in \left[0,\frac{\pi }{2}\right]$ are added relative positional encodings. Note that all of our positional encoding for keys, queries and values are generated randomly and independently. The ablation study will show the effectiveness of our proposed positional encoding.

### Loss function

In this study, we adopt a weighted soft Jaccard index approximation (WSJA) loss [5]. The soft Jaccard index *SJA*_*i*_ for the class *i* is calculated by

$$SJA_{i}=\frac{\sum \left(y_{i}\;\times \;{\hat{y}}_{i}\right)+\varepsilon }{\sum y_{i}+\sum {\hat{y}}_{i}-\sum \left(y_{i}\;\times \;{\hat{y}}_{i}\right)+\varepsilon },i=1,...,5,$$ (13)

where ε=1e−3 is a smoothing constant, and $y_{i},{\hat{y}}_{i}$ are GT and predicted label for the class *i*. Our loss function ℒ is accomplished by summing SJA over five classes and imposing weighted attention on the two classes of minimum SJA, which is indicated in Eq 14:

$$ℒ=-\text{sum}\left(\text{SJA}\right)-w_{1}\min\left(\text{SJA}\right)\;-w_{2}\min\left(\left\{\text{SJA}\backslash \min\left(\text{SJA}\right)\right\}\right),\text{SJA}=\{SJA_{i}{\}}_{i=1}^{5}.$$ (14)

The weights $w_{1},w_{2}$ are set to 1.0, 0.8 separately.

## Materials

### Dataset and metrics

We evaluated all models on the only public blastocyst dataset [53], which consists of 249 blastocyst images and their masks. There are five classes in the dataset: ZP, TE, ICM, blastocoele and background. We have to point out that the dataset version used in Blast-Net [5] only consists of 200 images, and they did not provide criteria for dividing the training set and test set. Therefore, we randomly selected 199 images from the blastocyst dataset as our training set while the remaining 50 images constitute the test set.

To evaluate the performance of the network, five evaluation metrics are commonly used [53]: Accuracy, Precision, Recall, Dice Coefficient (Dice), and Jaccard Index (Jaccard), which are defined as:

$$\text{Accuracy}=\frac{TP+TN}{TP+FP+TN+FN},$$ (15)

$$\text{Precision}=\frac{TP}{TP+FP},$$ (16)

$$\text{Recall}=\frac{TP}{TP+FN},$$ (17)

$$\text{Dice}=\frac{2\;\times \;TP}{2\;\times \;TP+FP+FN},$$ (18)

$$\text{Jaccard}=\frac{TP}{TP+FP+FN}.$$ (19)

These indicators are defined based on four parameters: TP (true positive), FP (false positive), TN (true negative), and FN (false negative).

### Implementation details

We conducted training for 2000 epochs with specific hyperparameters: an initial learning rate of 0.0008, momentum set to 0.9, and weight decay of 0.0005. To adaptively adjust the learning rate, we employed the Poly learning rate policy. Network parameter updates were performed using the Adam optimizer [54]. Our implementation utilized PyTorch (version ≥ 1.10), and training as well as testing were executed on a 32GB NVIDIA Tesla V100 GPU. Prior to inputting images into the models for both training and testing, we standardized their dimensions to 256×256. Furthermore, during the training stage, each sample underwent augmentation, including random scaling, rotation, cropping, and horizontal flipping.

## Experimental results

### Comparison with state-of-the-art methods

The baselines we compared include two types: CNN-based methods, such as U-Net [29], AttUNet [30], Unet++ [31], Pranet [33], nnUnet [34], Deeplabv3+ [8], Unet3+ [55], FCN [56], PSPNet [57], DANet [58], CCNet [59], Blast-Net [5], PCS-Net [60] and SPC-Net [61]; and Transformer-based methods, such as SETR [46], TransUNet [47], Segtrans [48], Swin-Unet [62], UCTransnet [63], and MedT [49]. The baselines are trained by applying the same binary cross-entropy (CE) loss between the prediction and the ground truth. To ensure fairness, we trained the whole comparison methods involved in this paper with the same configuration.

#### Quantitative results.

To quantitatively evaluate the embryo segmentation performance, five metrics including Accuracy, Precision, Recall, Dice, and Jaccard are tabulated in Table 1. We achieved the best performance of Accuracy, Precision, Recall, Dice, and Jaccard with 93.86%, 91.81%, 92.25%, 92.02%, and 85.45% respectively. Note that the authors of Blast-Net do not provide their training codes. Thus, we reproduced their method and trained it on the training set.

**Table 1:** Quantitative results in comparison with the comparison methods. “ ” and “**” denote significance levels of p≤0.05 and p≤0.01, respectively, from a two-sided paired t-test when comparing our method with others. The best results are highlighted in bold.

Category	Methods	Accuracy (%)	Precision (%)	Recall (%)	Dice (%)	Jaccard (%)
CNN-based	U-Net [29]	93.53$\pm 0.05^{**}$	91.43$\pm 0.11^{*}$	91.78$\pm 0.05^{**}$	91.59$\pm 0.05^{**}$	84.74$\pm 0.08^{**}$
	Blast-Net [5]	93.35$\pm 0.12^{**}$	91.07$\pm 0.19^{**}$	91.60$\pm 0.22^{*}$	91.33$\pm 0.18^{*}$	84.32$\pm 0.30^{*}$
	AttUnet [30]	93.55$\pm 0.05^{**}$	91.37$\pm 0.09^{**}$	91.88$\pm 0.05^{*}$	91.62$\pm 0.05^{**}$	84.78$\pm 0.08^{**}$
	Unet++ [31]	93.50$\pm 0.07^{**}$	91.13$\pm 0.26^{*}$	91.99$\pm 0.09^{*}$	91.54$\pm 0.09^{**}$	84.64$\pm 0.16^{**}$
	nnUnet [34]	93.63$\pm 0.02^{**}$	91.59$\pm 0.12^{**}$	91.82$\pm 0.04^{**}$	91.70$\pm 0.04^{**}$	84.92$\pm 0.06^{*}$
	Pranet [33]	93.51$\pm 0.07^{**}$	91.23$\pm 0.08^{**}$	91.83$\pm 0.15^{*}$	91.52$\pm 0.11^{**}$	84.64$\pm 0.18^{**}$
	Unet3+ [55]	93.43$\pm 0.04^{**}$	91.21$\pm 0.14^{**}$	91.76$\pm 0.08^{**}$	91.48$\pm 0.04^{**}$	84.55$\pm 0.06^{**}$
	Deeplabv3+ [8]	93.62$\pm 0.02^{**}$	91.42$\pm 0.08^{**}$	91.97$\pm 0.10^{*}$	91.68$\pm 0.03^{**}$	84.90$\pm 0.04^{**}$
	FCN [56]	93.51$\pm 0.02^{**}$	91.34$\pm 0.04^{**}$	91.90$\pm 0.08^{*}$	91.62$\pm 0.03^{**}$	84.77$\pm 0.06^{**}$
	DANet [58]	93.42$\pm 0.03^{**}$	91.14$\pm 0.10^{**}$	91.86$\pm 0.04^{**}$	91.50$\pm 0.05^{**}$	84.57$\pm 0.08^{**}$
	CCNet [59]	93.60$\pm 0.02^{**}$	91.41$\pm 0.05^{*}$	92.09±0.07	91.75$\pm 0.02^{**}$	84.98$\pm 0.04^{**}$
	PSPNet [57]	93.31$\pm 0.03^{**}$	90.94$\pm 0.09^{**}$	91.52$\pm 0.07^{**}$	91.23$\pm 0.07^{**}$	84.13$\pm 0.11^{**}$
	PCS-Net [60]	93.30$\pm 0.03^{**}$	91.12$\pm 0.17^{*}$	91.45$\pm 0.21^{*}$	91.27$\pm 0.03^{**}$	84.19$\pm 0.06^{**}$
	SPC-Net [61]	93.20$\pm 0.10^{**}$	90.93$\pm 0.19^{**}$	91.46$\pm 0.07^{**}$	91.19$\pm 0.12^{**}$	84.04$\pm 0.19^{**}$
Transformer	Segtrans [48]	93.56$\pm 0.03^{**}$	91.36$\pm 0.16^{*}$	92.03$\pm 0.16^{*}$	91.69$\pm 0.04^{**}$	84.88$\pm 0.06^{**}$
	UCTransnet [63]	93.55$\pm 0.04^{**}$	91.26$\pm 0.05^{**}$	92.04±0.08	91.64$\pm 0.02^{**}$	84.81$\pm 0.04^{**}$
	MedT [49]	93.05$\pm 0.03^{**}$	90.59$\pm 0.07^{**}$	91.22$\pm 0.10^{**}$	90.90$\pm 0.08^{**}$	83.59$\pm 0.13^{**}$
	SETR [46]	91.79$\pm 0.08^{**}$	89.05$\pm 0.16^{**}$	89.59$\pm 0.12^{**}$	89.31$\pm 0.10^{**}$	81.05$\pm 0.15^{**}$
	Swin-Unet [62]	92.71$\pm 0.32^{*}$	90.10$\pm 0.38^{**}$	90.85$\pm 0.56^{*}$	90.46$\pm 0.48^{*}$	82.91$\pm 0.76^{*}$
	TransUNet [47]	93.48$\pm 0.05^{**}$	91.23$\pm 0.07^{*}$	91.82±0.28	91.52$\pm 0.12^{**}$	84.61$\pm 0.20^{**}$
**Ours**	**BTFormer**	**93.86** ±0.03	**91.81** ±0.08	**92.25** ±0.03	**92.02** ±0.04	**85.45** ±0.06

As shown in Table 1, the Blast-Net’s effect is unsatisfactory, with only 84.32% Jaccard. The nnUnet increases the information of multi-scale supervision, with Jaccard reaching 84.92%. However, the Jaccard using the classic attention methods of DANet and CCNet only reaches 84.57% and 84.98%, respectively. Some transformer-based methods employed with transformer backbones (such as ViT) lead to worse results. It means that these transformer models need to be trained on large-scale datasets, which is rare for medical image analysis. The Segtrans achieves a good result because it applies CNNs as a backbone. MedT does not require large-scale datasets to train but lacks proper performance. Our BTFormer can handle this small-scale embryo dataset and outperform both CNN-based and Transformer-based methods.

#### Qualitative results.

**Fig 5:**
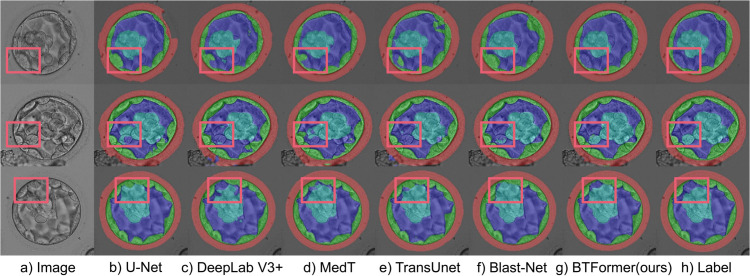
Qualitative comparison of BTFormer and the state-of-the-art models. The red boxes depict regions where BTFormer recognizes better than others with broader perspectives.

For qualitative analysis, we visualize the predictions for our BTFormer and other five representative approaches including CNN-based methods (U-Net, DeepLabV3+, and Blast-Net) and Transformer-based methods (TransU-Net and MedT) as revealed in Fig 5. To express the results vividly, we use different colors to represent different components in blastocyst images. The first column is the input blastocyst images and the last column is their corresponding labels. The remaining columns are predictive results of different approaches.

As exhibited in Fig 5, for some special regions easily confused (perhaps due to the local inconsistency), other methods cannot take full advantage of the global relationship and make a wrong decision. However, our model can still figure out its right label. As seen in Fig 2(e), due to our axial-free attention, we have diversified cues across the whole image. This design makes our prediction more accurate as they can understand a region from a non-local view.

#### Computational efficiency.

**Fig 6:**
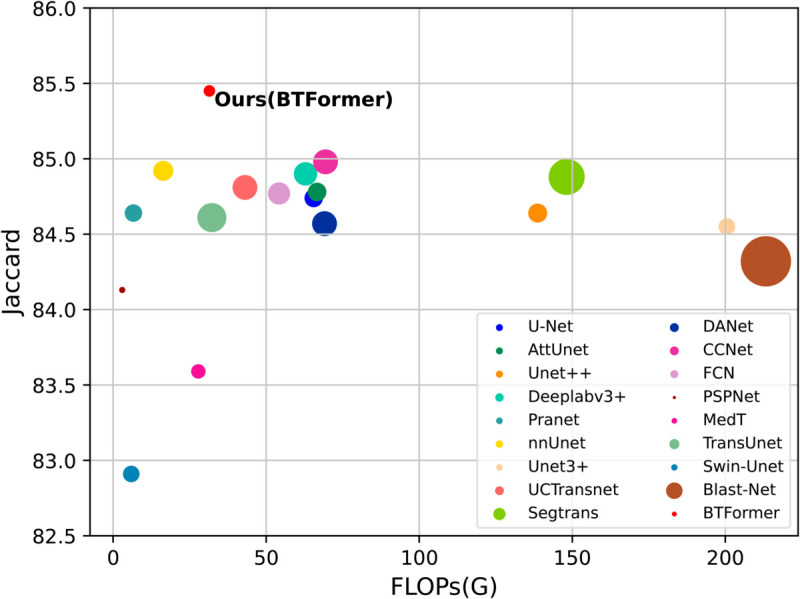
The parameter number and FLOPs of different models. The size of a circle relatively represents the number of parameters. The larger area one circle covers, the heavier computation a model has.

Fig 6 gives the parameter numbers and FLOPs of several representative methods. In general, Transformer-based models require more computation and GPU RAM than CNN-based methods. However, in Fig 6, our method presents competitive computation efficiency. Due to the sparse adaptation of self-attention *i.e.* axial-free attention, BTFormer leverages smaller computation costs while maintaining global connections.

### Ablation studies

Several ablation studies were conducted and the settings of them were variants of the standard one. Dice and Jaccard are applied in our ablation studies, as the trends in other indicators are the same.

#### Selection of attention layer number.

**Fig 7:**
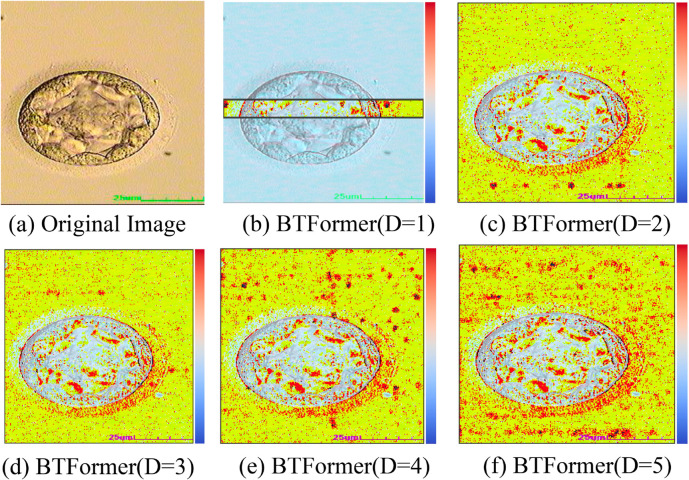
Effective receptive fields (colored dots) of BTFormer with different D on a 256×256 input image. As *D* increases, we can obtain more widespread receptive fields with less concentrated focus in certain directions.

The selection of the number *D* in Eq 1 is of great essential to form the axial-free attention block. First, we try to probe the influence of the receptive field with various *D*, which is depicted in Fig 7. When *D* is equal to 1, the model only acquires the narrow effective receptive field along the width axis as seen in Fig 7(b). The axial-free attention layers will become crossed if *D* = 2. Under this condition, BTFormer hence gathers dense attention both from the width axis and the height axis as shown in Fig 7(c). As the number of *D* rises, the serried dots grow dispersed and filled with the whole image. It means that a larger *D* leads to garnering global representations and a vast receptive field.

**Fig 8:**
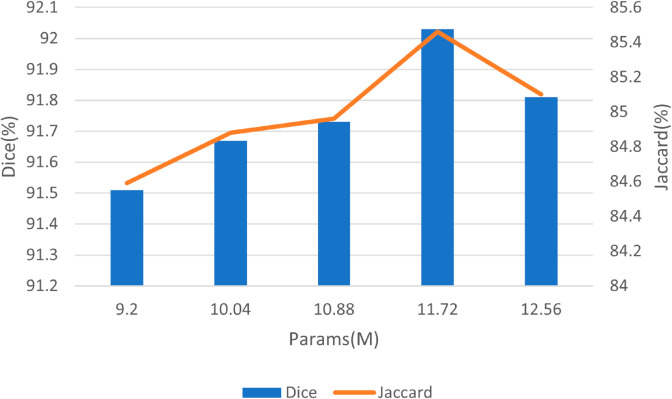
Performance comparison of the number of different axial-free attention layers. From left to right, D corresponds to 1-5.

Then, comparisons of Dice and Jaccard for different *D* are studied. Fig 8 shows the Dice and Jaccard results of number *D* varying from 1 to 5. As we can see from Fig 8, BTFormer with a larger number *D* occupies more computation. Thus *D* can not be too large on account of computational efficiency. On the other hand, BTFormer (*D* = 5) behaves better than BTFormer (*D* = 3) and BTFormer (*D* = 1). However, a larger number of *D* does not always ensure good performance. BTFormer (*D* = 4) outperforms the model BTFormer (*D* = 5). Observing both of these two conditions collects long-distance information along the width axis and the height axis, a possible explanation is that the two vertical directions occupy the main features compared with other directions. Specifically, BTFormer (*D* = 4) is the best choice with the highest Dice and Jaccard and a lower computation cost.

#### The impact of learnable sinusoidal relative positional encoding.

And Table 2 compares learnable sinusoidal relative positional encoding with the original learnable relative positional encoding (LRPE) schemes and no positional encoding (PE). We evaluate our BTFormer without any other changes in the training settings except the PE scheme.

**Table 2:** The effect of different types of position encoding. We compared the performance of BTFormer with position encoding and without position encoding. “ ” and “**" denote significance levels of p≤0.05 and p≤0.01, respectively, from a two-sided paired t-test when comparing our best model with others. The best results are shown in bold.

Positional Encoding Type	Dice (%)	Jaccard (%)
No PE	91.62$\pm 0.14^{*}$	84.79$\pm 0.23^{*}$
LRPE	91.74$\pm 0.04^{**}$	84.98$\pm 0.07^{**}$
LSRPE (Ours)	**92.02** ±0.04	**85.45** ±0.06

It appears that Table 2 depicts the effectiveness of our proposed LSRPE where BTFormer with LSRPE achieves the best performance. From Table 2, we also observe that the performance of BTFormer without position encoding will degrade.

#### The type of aggregation.

Table 3 compares our soft aggregate operation with four common aggregation methods to condense the non-local dependencies obtained by axial-free attention layers from a given direction. Here, ‘Add’ means we add features directly and ‘Wadd’ is the add operation with learnable weights on outputs. ‘Cat’ notes that features are concatenated together and then pass a 1×1 convolution to keep the output channels, which is the same with the right branch in Fig 3(c). The left branch of Fig 3(c) is denoted as ‘Agg’ in Fig 3(c). ‘Soft Agg’ in the last row is our proposed soft aggregation operation.

**Table 3:** Different aggregation ways. We have designed four aggregation approaches to fuse the outputs of axial-free attention layers, which are common in other network architectures. “**" denote significance levels of p≤0.01, from a two-sided paired t-test when comparing our best model with others. The best results are shown in bold.

Aggregation Method	Dice (%)	Jaccard (%)
Add	91.84$\pm 0.05^{**}$	85.16$\pm 0.08^{**}$
Wadd	91.86$\pm 0.06^{**}$	85.18$\pm 0.10^{**}$
Cat	91.76$\pm 0.06^{**}$	85.03$\pm 0.09^{**}$
Agg	91.81$\pm 0.05^{**}$	85.10$\pm 0.07^{**}$
Soft Agg (Ours)	**92.02** ±0.04	**85.45** ±0.06

Table 3 shows the effectiveness of our proposed soft aggregation operation. A possible explanation is that the importance of semantic information from different directions is quite biased. As seen in Table 3, the direct add operation achieves unsatisfactory performance, which treats the features equally. Thus, when we apply a simple weighted add, the performance is improved. ‘Agg’ is a complex weighted add and behaves well when ‘Cat’ regards the outputs as one feature. We achieve the best performance with soft aggregation.

#### The impact of supervision.

Table 4 compares the BTFormer with supervision and without supervision. We evaluate our BTFormer and keep the same configures except the supervision scheme. Table 4 depicts the importance of deep supervision, which is implemented with multi-scale levels.

**Table 4:** Dice and Jaccard index change with/without supervision. “**" denote significance levels of p≤0.01, from a two-sided paired t-test when comparing our best model with others. The best results are shown in bold.

Method	Dice (%)	Jaccard (%)
w/o supervision	91.80$\pm 0.06^{**}$	85.09$\pm 0.10^{**}$
w/ supervision (Ours)	**92.02** ±0.04	**85.45** ±0.06

#### The effect of loss function.

To investigate the role of our WSJA loss, we trained our BTFormer employed with two loss functions. In Table 5, ‘CE’ denotes binary cross-entropy loss. ‘SJA’ denotes the soft (*i.e.* differentiable) Jaccard index approximation loss with only one weight in the class of minimum SJA:

**Table 5:** Performance with different loss functions. “ ” and “**” denote significance levels of p≤0.05 and p≤0.01, respectively, from a two-sided paired t-test when comparing our best model with others. The best results are shown in bold.

Method	Dice (%)	Jaccard (%)
CE	91.79$\pm 0.10^{*}$	85.09$\pm 0.17^{*}$
SJA	91.74$\pm 0.02^{**}$	84.98$\pm 0.04^{**}$
WSJA (Ours)	**92.02** ±0.04	**85.45** ±0.06

$${ℒ}_{SJA}=-\text{sum}\left(\text{SJA}\right)-\min\left(\text{SJA}\right),\text{SJA}=\{SJA_{i}{\}}_{i=1}^{5}.$$ (20)

## Conclusions

In this paper, we introduced a Blast-transformer (BTFormer) for the semantic segmentation of the main four components of the human blastocyst. This framework is built upon the novel axial-free attention layer, which effectively leverages two key image features: local inconsistency and rotation consistency. Furthermore, we enhanced our transformer layers with a novel positional encoding and an improved soft aggregation operation. The experimental results show that our method significantly improves the segmentation performance of various blastocyst tissues and expands the receptive field. Compared with existing state-of-the-art methods, BTFormer achieves superior segmentation performance across five metrics while maintaining competitive efficiency.

While BTFormer achieves robust performance in standard blastocyst morphologies, its accuracy may decrease in edge cases (*e.g.* fragmented ICM, oblique imaging). In the future, we will integrate multi-focal plane data or uncertainty quantification to mitigate this. In addition, the scarcity of publicly available datasets highlights the urgent need for robust validation methodologies tailored for multi-source data. Therefore, future research should also focus on maximizing the use of unlabeled data to significantly improve model performance beyond current capabilities.
